# New Functions of Arthropod Bursicon: Inducing Deposition and Thickening of New Cuticle and Hemocyte Granulation in the Blue Crab, *Callinectes sapidus*

**DOI:** 10.1371/journal.pone.0046299

**Published:** 2012-09-28

**Authors:** J. Sook Chung, Hidekazu Katayama, Heinrich Dircksen

**Affiliations:** 1 Institute of Marine and Environmental Technology, University of Maryland Center for Environmental Science, Baltimore, Maryland, United States of America; 2 Department of Zoology, Stockholm University, Stockholm, Sweden; University of Rouen, France

## Abstract

Arthropod growth requires molt-associated changes in softness and stiffness of the cuticle that protects from desiccation, infection and injury. Cuticle hardening in insects depends on the blood-borne hormone, bursicon (Burs), although it has never been determined in hemolymph. Whilst also having Burs, decapod crustaceans reiterate molting many more times during their longer life span and are encased in a calcified exoskeleton, which after molting undergoes similar initial cuticle hardening processes as in insects. We investigated the role of homologous crustacean Burs in cuticular changes and growth in the blue crab, *Callinectes sapidus*. We found dramatic increases in size and number of Burs cells during development in paired thoracic ganglion complex (TGC) neurons with pericardial organs (POs) as neurohemal release sites. A skewed expression of Burs β/Burs α mRNA in TGC corresponds to protein contents of identified Burs β homodimer and Burs heterodimer in POs. In hemolymph, Burs is consistently present at ∼21 pM throughout the molt cycle, showing a peak of ∼89 pM at ecdysis. Since initial cuticle hardness determines the degree of molt-associated somatic increment (MSI), we applied recombinant Burs *in vitro* to cuticle explants of late premolt or early ecdysis. Burs stimulates cuticle thickening and granulation of hemocytes. These findings demonstrate novel cuticle-associated functions of Burs during molting, while the unambiguous and constant presence of Burs in cells and hemolymph throughout the molt cycle and life stages may implicate further functions of its homo- and heterodimer hormone isoforms in immunoprotective defense systems of arthropods.

## Introduction

The cuticle protects arthropods from desiccation or water influx, injury, infections by microorganisms, moulds shape and stability of the body and determines growth. As a complex secretion product of an underlying hypodermis, this non-living structure varies along the body and undergoes molt-stage dependent morphological and structural changes from being thin and soft to thick and hard. Its rigidity finally supports a stable exoskeleton but restricts continuous somatic growth. Therefore, production of new cuticle prior to shedding of the old one, the ecdysis process, followed immediately by stretching to final size and hardening of the new cuticle are essential steps for the typically discontinuous growth in all arthropods.

Wing expansion and tanning as the final steps of insect growth via molting and ecdysis are known to be regulated by Bursicon (Burs) [1], [2], [3], [4]. A hormonal action of Burs was inferred several decades ago through ligature and transplantation bioassays, resulting in phenotypical responses of the maturation of wings through cuticle tanning and cuticle sclerotization [5], [6]. Only recently, the structure of *Burs* which consists of a heterodimer of Burs α and ß subunits (1∶1) forming a typical cystine knot protein, its mode of action [7], and its receptor LGR2 were identified [8], [9]. The target cells are still unknown [8], [9], although proposed neurohemal release from muscle terminals corroborated humoral actions [10]. In addition, the involvement of various biphenols, tyrosine hydroxylase (TH), dopa-decarboxylase (DDC), N-acetyl-transferase and phenoloxidases (PO) in tanning [3], [10], [11], [12], [13], [14], [15] suggests that this biological process is rather complex in arthropods.

In crustaceans, mechanisms and hormonal control regulating somatic growth via cuticle tanning and hardening are poorly explored, except for some early reports on phenolic tanning activity in newly formed cuticle, epidermis and hemocytes [16], [17]. However, it is well established that optimal somatic growth requires water uptake driven by crustacean hyperglycemic hormone (CHH) during ecdysis followed by a tightly scheduled tanning process [18]. Recently, the thoracic ganglia complex (TGC) including subesophageal, thoracic and abdominal neuromeres of the shore crab *Carcinus maenas* and the European lobster, *Homarus gammarus* was identified as the sole site for *Burs* expression [19], [20]. An unequal expression of Burs ß versus Burs α indicative of the possible existence of Burs ß homodimers was noted in the shore crab TGC [19], a phenomenon first described in moth but only using immunohistochemistry [21]. Nonetheless, an integrative systems approach to show sites and pattern of release of identified dimeric Burs proteins as true hormones circulating in the hemolymph within the functional context of cuticle-associated tissues involved in hardening and somatic growth and during development has never been undertaken in any arthropod.

Here, we report identification of CasBurs protein hormones and neurohemal pericardial organs (POs) as their storage and release sites in the blue crab, *Callinectes sapidus*. The POs originate in neurosecretory neurons of the TGC as their site of synthesis. Using hatchery-raised animals with clearly tractable life stages, we found that CasBurs cells undergo dramatic changes in sizes and numbers during the animal’s molt and life cycles. However, to our surprise, CasBurs is consistently present in hemolymph throughout molt cycle and life stages, showing transient peak concentrations around completion of ecdysis and shortly after. More importantly, a skewed expression of CasBurs ß vs. α in the TGC results in formations of both a heterodimer of CasBurs ß plus α ( = CasBurs) and a homodimer of CasBurs ß in the PO. Moreover, we present the first evidence in crustaceans for functional roles of CasBurs in identified target tissue and associated cells of the cuticle: The hormone controls the deposition of epicuticle and exocuticle by the hypodermis during premolt, cuticle sclerotization during post-ecdysis, and granulation in cuticle-associated hemocytes.

## Results

### Identification and Expression of CasBurs α and ß and Proteins

The cloned cDNAs encode 121 amino acids (aa) long CasBurs α (EU677191) and 115 aa long CasBurs ß (EU677190) protein hormones after predicted cleavage of 27 aa signal peptides in both cases. Both subunits are very similar in primary structures among portunid crab species, as they differ only by few aa, resulting in 99% (α) and 94% (ß) sequence identity (Figs. S1A, S1B). When comparing the CasBurs proteins with those of the more diverged bursicon subunits from the branchiopod crustacean *Daphnia arenata* and the insect *Drosophila melanogaster*, the Burs α subunits appear to be more conserved (71% and 62%, respectively) than the Burs ß subunits (61% and 56%, respectively, Figs. S1A, S1B **)**.

**Figure 1:**
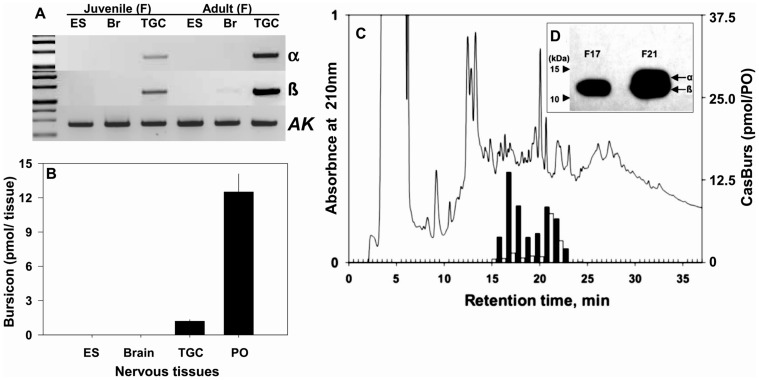
CasBurs synthesis in TGCs and its storage in the POs, and identification of CasBurs heterodimers and CasBurs ß homodimers in POs. (**A**) *CasBurs α* and *β* (20 ng total RNA tissue equivalents for RT-PCR assay) is expressed only in TGCs but not in ES or brains of a juvenile (∼50 mm CW) and an adult female (F; >125 mm CW) in intermolt, whilst arginine kinase (*AK*) served as control. (**B**) About ten times more CasBurs is stored in POs compared with TGCs (means ± SEM pmol/tissue (n = 6) estimated by CasBurs RIA; crab stage 15–16: 70–90 mm carapace width, CW). (**C**) CasBurs RIA identifies two major peak fractions after RP-HPLC separations of extracts of juvenile and adult POs (Chromatogram shown for juvenile POs only; *closed bars*: contents of juvenile POs at E_0%_ stage; *open bars*: adult females at C_4_ stage). (**D**) Western blot analysis of fractions F17 and F21 obtained from juvenile POs clearly showing the CasBurs ß subunits only in F17 and CasBurs ß and α subunits in F21 (Marker *arrowheads* at 15 and10 kDa).

Identified CasBurs α and ß as obtained from single specimens both show stronger expression in TGCs of adult than in TGCs of young juvenile females, while arginine kinase (*AK*) expression, that was used as a reference, house keeping gene, remains constant. CasBurs α and ß is not detected in brain or eyestalk (ES) tissues (Fig. 1A). These expression data are corroborated by contents of corresponding CasBurs protein hormone subunits as measured in ES, brain, TGC, and PO of crabs (from 17th or 19th crabs) with a specific and sensitive radioimmunoassay (RIA; EC_50_ value at 29.1±2.3 pM; n = 8; detection limit of ≤2.3 pM, Fig. S5). CasBurs is again not detected in brain or ES samples by RIA. However, POs contain ten times higher amounts of CasBurs (∼12.5±1.6 pmol CasBurs/PO, n = 10) than TGCs (1.2±0.1 pmol/TGC, n = 10; Fig. 1B). CasBurs contents in POs of juveniles at E_0%_ are higher than in POs of adult females at intermolt C_4_, and split into two major CasBurs-immunoreactive (-ir) fractions (F) after RP-HPLC separation as identified by the Burs RIA. Highest amounts of CasBurs-ir are detected in F17 (13.5 pmol/PO) of juveniles and F21 (8.4 pmol) of juveniles at premolt and F21 of adult females at intermolt (7.4 pmol) (Fig. 1C). Western blot analysis after SDS-PAGE under reducing conditions reveals F21 of juveniles as consisting of two CasBurs-ir bands located between the 10 and 15 kDa markers, whilst F17 of juveniles contains only one single band of the same molecular weight as the lower band in F21 (Fig. 1D). This indicates the presence of both CasBurs ß and the slightly larger CasBurs α in F21 but CasBurs ß only and no CasBurs α in fraction F17.

### Changes in CasBurs Cells in TGC during Postembryonic Life Stages

**Figure 2:**
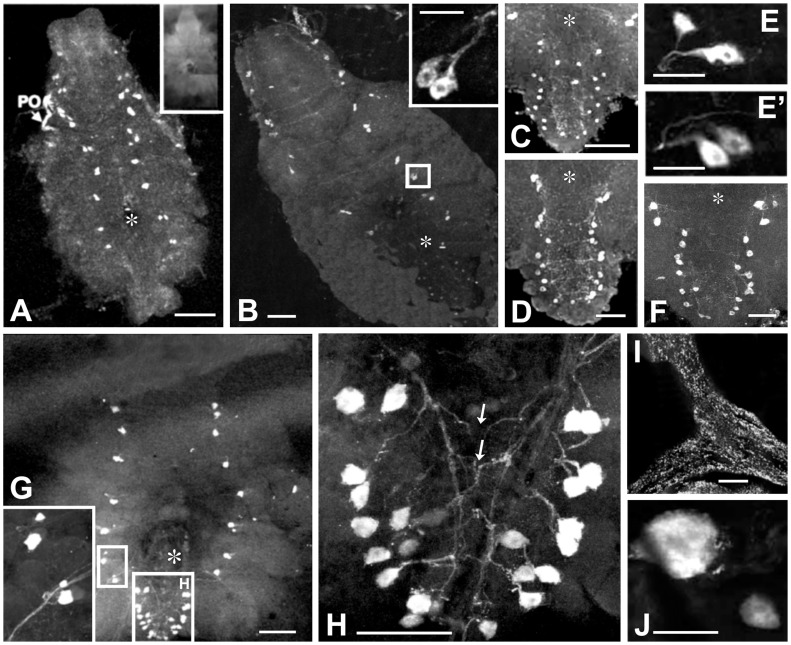
Immunolocalization of two different types of bilaterally paired CasBurs neurons per neuromere in crab TGC and POs during late postembryonic development. (**A**) CasBurs neurons in the subesophageal and thoracic but not in the abdominal neuromeres of TGC of a postlarva, megalopa. Inset shows a pre-absorption control of an adult TGC. (**B**) CasBurs neurons in the entire TGC of the 1^st^ crab. (**C–F**) Details of neurons in the last thoracic and abdominal neuromeres of the 1^st^(**C**), 3^rd^ (**D**), 4^th^ crab (**F**). (**E**, **E’**) Two distinct types of CasBurs neurons in thoracic segments showing a difference in staining intensity but no size disparity yet. (**G**) CasBurs neurons in the TGC of a 12^th^ crab, inset: enlarged cells in the 4^th^ and the 5^th^ thoracic neuromeres; note clear disparity in size of the distinct neuron types in abdominal neuromers. (**H**) Higher magnification of abdominal neuromeres of the 12^th^ crab’s TGC without apparent size disparity; note the contralateral projection patterns (arrows). (**I**) Fibers and terminals in the anterior bar of a PO (**J**) A pair of typically disparate-sized thoracic cell bodies in an adult TGC. *Asterisks*: foramen of the sternal artery. Whole mounts in all cases; scale bars A, B, C, D, F, I = 100 µm; B inset, E, E’ = 50 µm; H = 200 µm; J = 25 µm.

Whole mount immunohistochemistry demonstrates that up to 50 contralaterally projecting lateral neurons in total in the TGC synthesize CasBurs and project to axon endings in the POs as its storage and release sites (Fig. 2). All CasBurs neurons in the entire TGC of *C. sapidus* co-localize CCAP (Fig. S2A, B–B”), and pre-absorption controls show no staining of Burs cells (inset to Fig. 2A). Characteristically, dramatic changes in numbers and sizes are observed for CasBurs/CCAP cells located in both thoracic and abdominal neuromeres of megalopa and the first crab stages. The latter stage involves a metamorphic molt, as the elongated abdomen of a megalopa folds back against the thorax to form the shorter abdomen of a crab. Whereas the abdominal neuromeres of megalopae do not yet show any CasBurs cells (Fig. 2A), a total of 24–25 cells with small sizes (<10 µm) appear in the abdominal neuromeres only in the 1^st^ crab stage (Figs. 2B, 2C). Furthermore, diameters of CasBurs cell bodies in thoracic neuromeres increase significantly in sizes from ∼10 µm in the megalopa to ∼15 µm in the 1^st^ crab (Fig. 2B) and to 20–25 µm at the 4^th^ crab (Figs. 2E, 2E’). Sizes and projection patterns of these cells remain just as large (∼10 µm at the 3^rd^ and 10–12 µm at the 4^th^ crab, Figs. 2D, 2F, respectively) and densely stained as other CasBurs neurons in the subesophageal and thoracic neuromeres until the 12^th^ crab (∼50 µm, Fig. 2G). However, the paired CasBurs cells located bilaterally in the five thoracic leg neuromeres appear equal in size in the megalopa, the 1^st^ crab, and the 4^th^ crab (Fig. 2A, insets, Figs. 2B, 2E, 2E’); but at least from the 12^th^ crab onwards, these cells start to clearly display the typical size disparity of a large and a small type of CasBurs cells (inset to Fig. 2G) in all subesophageal and thoracic (Fig. 2G; large type 40–50 µm, and small type 20 µm), but not in the abdominal neuromeres (Figs. 2G, 2H). The large type cells project laterally and dorsally to release sites in the POs, whereas the small type cells are descending interneurons (Fig. 2I). This pattern persists into the adult stage (Fig. 2J; large type 25–30 µm, small type∼10 µm). Thus, among the total of approximately 50 CasBurs cell bodies in adult crabs, 8 subesophageal and 5 thoracic bilateral pairs of cells show different sizes of cell bodies whereas those of all 12–13 abdominal bilateral pairs display equal sizes.

### Dynamic Changes in CasBurs Expression, Protein Contents, and Release Patterns during the Molt Cycle

**Figure 3:**
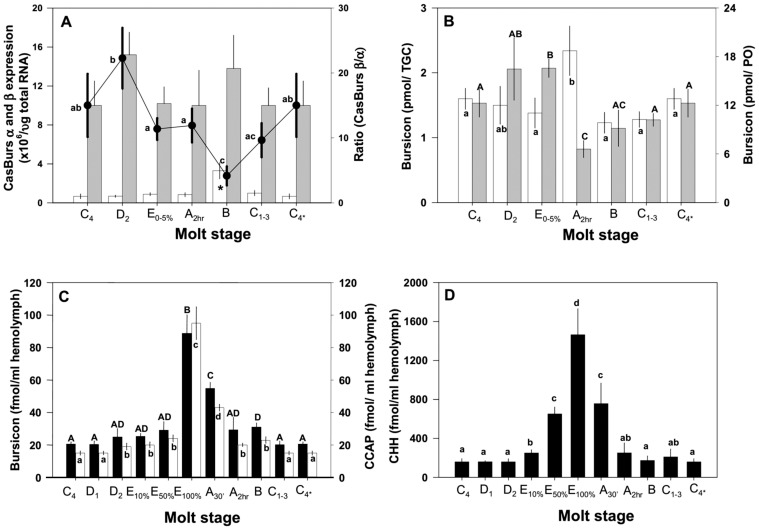
*CasBurs α* and *ß* expressions in TGCs and CasBurs, CCAP and CHH protein contents in TGCs, POs and hemolymph during the molt cycle. (**A**) The data of the QRT-PCR assays show copies/µg of TGC total RNA (n = 6–12) of CasBurs α and CasBurs ß after normalization against those of *AK* (see Suppl. Information for details). *Open bars*: CasBurs α; *closed bars*: CasBurs ß. *Closed circles* represent the ratio of CasBurs ß vs. α. Statistical significance levels at *P*<0.05 (ANOVA, Tukey-Kramer multiple comparison test) as marked with *asterisk* or different letters for the ratio of CasBurs ß vs. CasBurs α. (**B**) Changes in the amounts of CasBurs protein in TGC (*open bars*) and PO (*closed bars*) during the molt cycle. Note the significant increase in TGC-contents and dramatic decrease in PO-contents during postmolt stage A_2 hr_. The data are shown as means ± SEM pmol/tissue (n = 8–15). Statistical significance (ANOVA, Tukey-Kramer multiple comparison test, *P*<0.05) is noted with capital letters for Burs in PO and lower case letters for Burs in TGC. (**C**) CasBurs (*closed bars*) and CCAP (*open bars*) contents measured in the same hemolymph samples from various molt stages show amounts close to each other at all sampling points. Concentrations are shown as means ± SEM pM hemolymph (n = 8–15). Statistical significance (ANOVA, Tukey-Kramer multiple comparison test, *P*<0.05) is indicated with different capital letters for Burs and different lower case letters for CCAP. (**D**) CHH titers of the same hemolymph samples as in C during the molt cycle. Note that significant increases of CHH titers are already beginning during early to mid (E_10%_–E_50%_) and late (E_100%_) ecdysis as indicated by lower case letters at *P*<0.05 (ANOVA, Turkey-Kramer multiple comparison test). Data are presented as means ± SEM pM (n = 8–15).

The expression of CasBurs ß (EU677190) in TGC (normalized against *AK*) as analyzed using QRT-PCR assays is consistent and almost unchanged during the molt cycle (Fig. 3A). CasBurs α expression remains relatively constant as well except during postmolt stage B, in which its expression is significantly increased (asterisk in Fig. 3A). CasBurs ß expression is always higher than that of CasBurs α, yielding ratios of CasBurs ß vs. α between 4 and 22. The highest ratio of ∼22 at premolt D_2_ stage was due to a slight but not significant increase in CasBurs ß expression, whereas the lowest ratio of 4 was caused by significantly elevated CasBurs α expression during post-molt stage B (Fig. 3A). This lowest ratio then gradually increased back to a steady state ratio of 16 during the intermolt stage.

The expression of CasBurs α and ß is reflected by the levels of CasBurs in the TGC and PO throughout the molt cycle, ranging from 1.2 to 2.3 pmol/TGC and from 6 to 18 pmol/PO, respectively (Fig. 3B). However, since an animal contains a pair of POs, the total amount of CasBurs per animal must be doubled, i.e. to maximally 36 pmol. Levels of CasBurs protein in the TGC are significantly higher during A_2 hr_ than during E_0–5%_, B, and C_1–3_ stages. However, the POs, which contain their largest amount of CasBurs (18 pmol/PO) during E_0–5%_, apparently had released their contents when attaining their lowest CasBurs content (6 pmol/PO) by the A_2 hr_ stage (Fig. 3B). The reduction of CasBurs in the PO during the ecdysis period between stages E_0–5%_ and postmolt A is paralleled by a dramatic surge in the hemolymph (Fig. 3C, closed bar), in which it reached a peak concentration at 89±11 pM only by E_100%_. This hemolymph level then precipitously drops to 55±4 pM at A_30 min_, eventually returning to the basal level of 29±8 pM at A_2 hr_ and remaining constant at 21±1 pM during the intermolt stage.

CasBurs cells are active in adults as shown by *CasBurs* mRNA expressions and immunohistochemistry in adult female TGCs (Figs. 1B inset, 2J). CasBurs contents in the hemolymph of both adult males and females amount to 15±1 pM (n = 8), which are significantly lower than those of juveniles as shown above. Since the observed co-localization of CCAP and CasBurs in immunopositive TGC neurons is indicative of co-release, we determined CCAP concentrations in the same samples using a sensitive and specific CCAP-RIA. The concentrations of CCAP in hemolymph and their pattern during the molt cycle are remarkably similar to those of CasBurs (Fig. 3C, open bars). CCAP and CasBurs are present at a ratio of 1∶1, and the highest concentration of CCAP at 95±10 pM occurs at E_100%_, which drops quickly to 43±2 pM at A_30 min_, and returns to basal levels of 15±1 pM by stage A_2 hr_. During intermolt, the concentration of CCAP is lower at 15±1 pM than that of CasBurs at 21±1 pM (*P*<0.05, Student’s *t* test; Fig. 3C).

Since the maximum of molt-associated somatic increment (MSI) depends on (i) swelling of the body by water uptake and (ii) cuticle hardening commencing in stage A (see below), we measured the hemolymph titers of the major inducer of the former, CHH [18], by a RIA [22] in the same samples. CHH titers show molt cycle-related changes in hemolymphs that are similar to those of CasBurs and CCAP, but at overall much higher concentrations. From basal levels such as 160±30 pM at D_2_ and 250±30 pM at E_0–5%_, i.e. at the beginning of ecdysis, thus rising somewhat earlier than CCAP and CasBurs, an elevation of CHH concentrations shows with 650±70 pM at E_50%_ (in mid-ecdysis) to peak concentrations of 1,498±300 pM at E_100%_. This elevated CHH level decreases slowly but significantly to 760±250 pM at A_30 min_ and returns to almost basal levels of 250±10 pM by stage A_2 hr_ (Fig. 3D).

### CasBurs Stimulates Thickening and Hardening of Cuticle and Hemocyte Recruitment and Granulation

**Figure 4:**
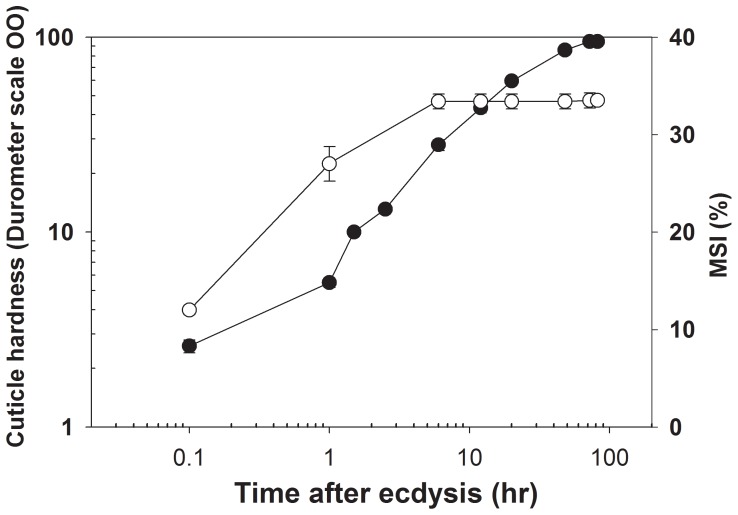
Initial cuticle hardness during the first 72 hr of post-ecdysis in *C. sapidus* determines the final molt-associated somatic increment (MSI). Cuticle hardness (*closed circles*) of the dorsal carapace after ecdysis as measured by a durometer (OO gauge). The MSI in percent (*open circles*) was calculated by measuring the carapace width of emerged animals versus that of their exuvia. The values are presented as means ± SEM (n = 8–20) as determined for crabs at 16th–18th crab stages (with 80–100 mm carapace width).

During ecdysis, the crab body is soft with values essentially around zero when measured by a gauge durometer (gauge OO), and is, thus, held mainly by hydrostatic pressure. The onset of cuticle hardening, which starts only shortly after ecdysis with very low durometer values of ∼6 after 6 min, is also of critical importance in determining the final somatic growth. We found that ∼ 70% of post-ecdysial MSI occurs already within ∼ 1 hr after ecdysis (Fig. 4), while the cuticle still remains soft and pliable.

**Figure 5:**
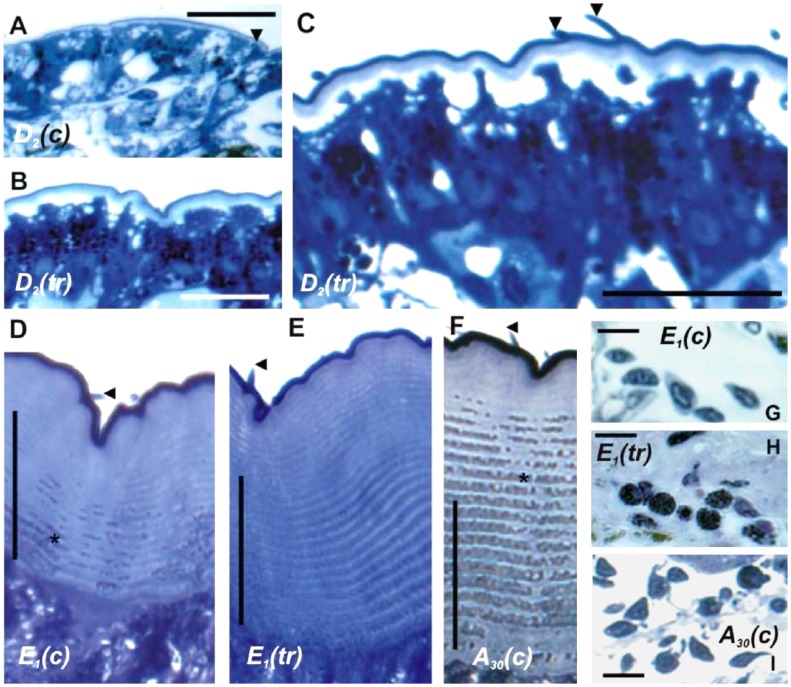
CasBurs drives deposition and hardening of cuticle and hemocyte granulation *in vitro*. Semi-thin cross-sections (1 µm) through pieces of dorsal carapace cuticle and hemocytes from control crabs (*c*; **A, D, F, G, I**) and after treatment with rCasBurs (*tr*, **B, C, E, H**). Control cuticle is thin in stage D_2_, but grows rapidly to considerable thickness already visible in ecdysis stage E_1%_ (**D**) and even more during stage A_30 min_ (**F**). Treatment (*tr*) with rCasBurs, however, clearly stimulates cuticle deposition already in the sensitive stage D_2_ (**B, C**) but also in stage E_1%_ (**E**). Hemocytes underneath the hypodermis change from virtually inactive as in E_1%_ and A_30 min_ controls (**G, I**) to highly active granulated appearance under rCasBurs treatment readily in stage E_1%._ (**H**). Scale bar: **A–C** = 25 µm, **D** and **E** = 50 µm, **F** = 100 µm **G–I** = 10 µm. *Arrowheads*: setae on the cuticle surface; *asterisk*: laminated layers of exocuticle.

Therefore, and since CasBurs hemolymph contents show peak values during late ecdysis and still elevated values about 30 min later at post-ecdysis, we tested pieces of newly laid carapace from the animals at late premolt (D_2_), early ecdysis (E_1%_) and postmolt (A_30 min_) stages by incubations with 1 µM rCasBurs for 30 min. The hormone-treated pieces show thicker cuticle than those from control animals (Figs. 5A–C). Epicuticle and exocuticle is notable in all control stages of D_2_, E_1%_, and A_30 min_, but there is no clear distinction between exo- and endocuticle in A_30 min_ (Figs. 5D, 5F). However, the cuticle of D_2_ stages incubated with rCasBurs is already significantly thicker (about 3.5 times; 5.9±0.3 µm, n = 10, *P*<0.01) than that of controls (1.7±0.2 µm, n = 12); Figure 5B. It is even furnished with protruding setae (marked by arrowheads in Fig. 5C) on the surface of the epicuticle indicative of being lifted by an increase in hydrostatic pressure (for expansion of the body) during and after ecdysis. Furthermore, at E_1%_ (Fig. 5D), the controls displayed thick (128.5±10.5 µm, n = 12) and homogeneously laminated cuticle (*asterisks* in Figs. 5D, 5F) newly laid at D_2_ (Fig. 5A). However, rCasBurs treated cuticle at E_1%_ shows significantly thicker exocuticle: 162±8.3 µm (1.3 times, n = 12, *P*<0.01) compared with controls (Figs. 5D, 5E).

At the beginning of ecdysis in controls (stage E_1%_, n = 15), a number of hemocytes occur underneath the hypodermis (Fig. 5G). However, hemocytes closely associated with rCasBurs-treated cuticle are heavily granulated at this stage (Fig. 5H). During postmolt at stage A_30 min_, most of these hemocytes do not contain granules anymore in the cytosol (Fig. 5I).

## Discussion

Our study provides the first evidence from a crustacean that arthropod Burs is a pleiotropic hormone with multiple targets that regulates (not only) the initial cuticle hardening process thereby determining molt-associated somatic growth. Such fundamental function of arthropod Burs is reflected by extraordinarily conserved amino acid sequences of CasBurs α and ß when compared to Burs protein sequences from both crustaceans and insects (Fig. S1). Furthermore, the coexistence of *CasBurs* α and ß protein hormones together with another highly conserved arthropod peptide CCAP within clearly homologous neuron types in crustaceans and insects [23], [24], strongly implies critical importance of the functional conservation of both hormones for molt control in arthropods.

*CasBurs* α and ß were found to be expressed exclusively in TGC, and CasBurs was detected immunochemically and by RIA in TGC and POs. Since all co-localized CasBurs/CCAP type-2 neurons have their cell bodies in the TGC and project to the POs [25], [26], [27], these TGC neurons are obviously the sites of synthesis of Burs and CCAP with the POs as their release site (Fig. 2, Fig. S2). A similar situation is found in insects [15] and has been predicted in *C. maenas* on the basis of *in situ* hybridization results [19]. We noted a distinct change in the sizes of the CasBurs cell bodies during the development of *C. sapidus*. At the early stages from the metamorphosis stage megalopa to the 12^th^ crab, each neuromere of TGC exhibits a pair of equal-sized CasBurs cell bodies. However, from the 12^th^ crab onwards, one cell body of the pair has become much larger than the other, as is well known for all pairs of neurons expressing CCAP in crabs [26], crayfish [28] and even insects [29], [30], [31]. Paired blue crab CasBurs/CCAP cells comprise the same cnc-type-2 neurosecretory neuron with projections into the PO and a cdn-type-1 interneuron as in all thoracic and abdominal neuromeres of adult shore crabs, *C. maenas* and crayfish *Orconectes limosus*, the cdn-type-1 cell body always being smaller than that of the cnc-type-2 neuron (Fig. 2I) [26]; the latter may imply that the hormonal release from cnc-type-2 neurons via POs becomes more important in the later stages of the crab. A similar size disparity is known for CCAP neurons of juvenile and adult crayfish [32] and likely reflects dramatic changes in neuronal activities during development and/or the molt cycle, but the underlying mechanisms and their functional significance are still unknown.

In fruit flies *D. melanogaster*, DrmBurs induces apoptosis in wing epidermal cells after the final ecdysis, i.e. the last metamorphic molt from pupae to adults [33], and the CCAP/Burs cells appear to die or shut off their production after adult eclosion [34]. Adult female *C. sapidus* cease molting after the pubertal-terminal molt and remain in an anecdysial intermolt stage whilst being in active reproductive phase [35]. However, blue crab CCAP/Burs cells continue to produce Burs during adulthood (Figs. 1A, 2J; see also below), their sizes become reduced to almost half the sizes for both cell types. This implies that Burs may have other functions that remains yet to be unraveled (see also below). Furthermore, a possible functional role of both peptides in the CasBurs/CCAP cdn-type 1 descending interneurons has not yet been studied in any crustacean, but may well be associated with the initiation of molting behavior as is well established in insects [29].

In insects, bursicon usually occurs as a heterodimer of Burs α and ß with LGR2 as its receptor [8], [9], [15], but the expression levels and patterns of *Burs* α and ß appear to be different in crustaceans and insects [19], [21]. Similar to the CCAP expression profiles in TGCs [25], the consistent expression of *CasBurs* in TGCs throughout development and all stages of the molt cycle changes only little with regard to CasBurs α in molt stage B and the ratio of CasBurs ß/α in stages D_2_ and B. The consistently high expression ratio of CasBurs ß/α can best be explained by the presence of CasBurs ß homodimer proteins in POs as firmly established here for the first time. This is most pronounced in juveniles (Fig. 1A; F17 in Fig. 1B). Therefore, it is tempting to propose the presence of a Burs subunit homodimer in addition to the Burs heterodimer, whenever a skewed ratio of *Burs* ß vs. α is found in a given species. It remains to be examined whether CasBurs homodimer ß and CasBurs heterodimer bind to a separate or the same receptor.

Levels of Burs proteins in TGC, POs and hemolymph show fluctuations during the molt cycle. PO contents increase from rather constant basal levels to only slightly higher amounts in premolt (D_2_) and during ecdysis, but drop dramatically at stage A_2 hr_, rendering the CasBurs levels to being highest in the TGC and lowest in the PO at this stage. Concomitantly, the CasBurs levels in POs show profiles exactly inverse to those in hemolymph, with CasBurs contents being highest in hemolymph and PO at the E_100%_ stage and lowest at stage A_2 hr_ (Fig. 3C). This clearly suggests that the largest amount of Burs is released during late ecdysis. All CasBurs levels return back to basal intermolt levels already by the following B stage. Of note, however, considerable basal levels of CasBurs (at 21±1 pM) are constantly present in hemolymph throughout the molt cycle including intermolt stage C_4_, as is observed throughout development from juvenile to adult stages.

The invariant release pattern for both peptides during the molt cycle clearly reflects the established co-localization of CasBurs and CCAP in all paired CCAP neuron-types (Fig. S2, Fig. 3). Interestingly, although hemolymph contents of CCAP are overall 10-fold higher in *C. maenas* than in *C. sapidus*, the release profiles of CCAP during the molt cycle are remarkably similar in both crabs [27]. Previous works have shown co-storage of CCAP and Burs in the same neurosecretory granules for Burs/CCAP cells the American cockroach, *Periplaneta americana,* and co-expression of CCAP and Burs mRNAs in TGC cells of the European lobster *H. gammarus* [20], [36]. However, we provide here the first evidence for concomitant release of both hormones from the same identified neurosecretory cells of the TGC and their neurohemal release sites in the POs.

Molt-stage specific expression of gut CHH (G-CHH) and its dynamic release into the hemolymph directly induce increased water uptake during ecdysis in *C. maenas* [18]. The same scenario regarding premolt-specific expression of G-CHH occurs in *C. sapidus* (J. S. Chung, unpublished results), although hemolymph levels of CHH during intermolt of adult *C. sapidus* are consistently ∼10 times higher than those measured in *C. maenas* [18], [22], [37]. This is indicative of *C. sapidus,* being very active swimmers, to have likely higher energy demands and to experience more diverse, dynamic and stressful environmental conditions than *C. maenas*. During intermolt, identical hemolymph samples contain 7- and 10-fold more CHH than CasBurs and CCAP. At completion of ecdysis (E_100%_), however, CHH contents rise to even higher values, 15–16 times than those of CasBurs and CCAP. Furthermore, CHH concentrations in the hemolymph also rise earlier and persist longer than those of CasBurs and CCAP (Figs. 3C, 3D).

These distinct profiles implicate the concerted and precisely timed involvement of all three hormones in the control of successful ecdysis and tanning processes. CHH being timely released in large amounts from the gut endocrine cells earlier than CCAP and CasBurs, i.e. already clearly elevated in E_50%_, induces first restricted, then, towards the end of ecdysis and directly thereafter, massive increases in water uptake through the gut [18], thereby facilitating body expansion to the final MSI (Fig. 4).

Body expansion by iso-osmotic uptake of water results in body size increments of 20–50% per molt in *C. sapidus* (see Fig. 4). A precisely balanced concentration of gut CasCHH in the hemolymph regulates the degree of MSI by water uptake [18], [38], [39], [40]. CCAP concomitantly drives the stereotyped behavior centrally to aid re-distribution of increasing hemolymph volume during and after ecdysis [41], [42], [43]. Hemolymph CasBurs, on the other hand, is critical in determining the duration and termination of MSI. Precise timing and duration of the occurrence of CasBurs in hemolymph delimits the extent of initial hardness and subsequent cuticle sclerotization to about 3 hr prior to mineralization which takes place within 3–5 hr after ecdysis [41]. This process is equivalent to insect wing expansion that is clearly regulated by Burs [15], as only the stretched surface area of the crab cuticle determines the degree of MSI. The timing of release and the hemolymph contents of these two physiologically active hormones gut CasCHH and CasBurs are thus extremely critical for an optimal MSI and, therefore, decisive for growth and survival in crustaceans. Interestingly, in the moth *Manduca sexta,* the closely CHH-related ion transport peptide (ITP) is also secreted at ecdysis [42], followed by known defined actions of CCAP and MasBurs during ecdysis and wing expansion the moth [29]. This implies that the genomic, precursor and primary structures [43], [44] as well as the functions of CHH and ITP in regulation of hemolymph water contents are conserved in arthropods. However, stimulation of water uptake in aquatic crustaceans [18] and water re-absorption in insects are not only a prerequisite for successful ecdysis, but must be finely tuned in concert with the actions of CCAP and Burs during ecdysis and post-ecdysis in order to ensure survival and growth of these arthropods.

For the first time, we directly examined the hormonal action of Burs on the cuticle and its underlying tissues that undergo morphological changes during the molt cycle [45] in more detail in an *in vitro* model. Normally, new cuticle becomes gradually thicker as shown in Figures 5A and 5D at D_2_ and E_1%_ control stages. However, even already after 30 min rCasBurs-treated cuticle becomes significantly thicker than control cuticle during these stages. Native Burs is known to be a glycoprotein in insects [15] but our rCasBurs is likely not glycosylated as it was produced in *E. coli*. For this reason, a relatively high concentration of 1 µM rCasBurs had to be employed for *in vitro* incubations of crab cuticle pieces, a concentration certainly much higher than its physiological levels as shown above. The surface of rCasBurs-treated newly laid cuticle was not smoothly extended as in controls but somewhat wavy, likely because of precocious hardening and lack of internal hemolymph pressure *in vitro* (Figs. 5B, 5C, 5E, 5F). Thus, in order to achieve the maximally possible final MSI (Fig. 4), the final cuticle hardening processes ought to commence only after a successful increase in surface area by water influx-driven body expansion has taken place. This result shows that a novel important hormonal action of Burs in crustaceans is the stimulation of rapid thickening of new cuticle including epi- and exocuticle during premolt as described here for the first time. Crustacean cuticle shows evenly distributed calcium already by 2 hr after ecdysis, as is brought about by the deposition of amorphous calcium carbonate into the exo- and endocuticle [41]. This means that this critical hormonal function of CasBurs in cuticle thickening is restricted to an extremely short window of <2 hr for postmolt changes in the cuticle, which is well corroborated by the timely peak concentrations of CasBurs (Fig. 3C).

In insects, prophenoloxidase (PPO) is present in hemocytes, hemolymph and cuticle and is involved in melanization and tanning processes [11], [12], [13], [14], [46], [47], [48]. Crustacean hemocytes exhibiting PPO activity [42], [43], [47] undergo changes in numbers and cell types during the molt cycle, in which granulocytes are major types from intermolt to premolt stages [46], whilst hyaline cells are predominant around ecdysis [49]. Hemocyte involvement in the tanning process during post-ecdysis is known for a long time [17], [46]. Cuticle-associated hemocytes such as granulocytes and hyaline cells rapidly attain much stronger granulation in the presence of rCasBurs than control hemocytes (Figs. 5G, 5H). We suggest that PPO residing in these hemocyte granules participates in the initial cuticle phenolic tanning process. Both, the cuticle-associated epidermal cells and certain granulated types of hemocytes [50] and likely hyaline cells [17] as well are thus novel target tissues of Burs.

In conclusion, somatic growth of aquatic crustaceans depends not only upon water uptake from the medium for the expansion of their body during and immediately after ecdysis but also critically on the properties of the new cuticle during and directly after ecdysis as well as throughout the life stages of arthropods. Newly laid pliable and water permeable linings of the gut as well as the new permeable cuticle are likely the primary sites for the massive but precisely controlled water intake slightly before, during and after ecdysis allowing for timely stretching and hardening of the cuticle as the new exoskeleton of the body. The result of our *in vitro*-experiments using rCasBurs acting on molt-stage defined cuticle pieces suggests that CasBurs displays a dual function in the deposition of new cuticle and its subsequent tanning and sclerotization. Burs stimulates (i) continued synthesis and accretion of novel ecdysial and post-ecdysial cuticle layers and (ii) their sclerotization. Furthermore, the consistent synthesis and presence in hemolymph of CasBurs throughout the molt cycle and the (iii) stimulation of PPO production in hemocytes as becomes apparent by their increased cytoplasmic granulation strongly suggests another important novel function of Burs in cuticle maintenance throughout the intermolt stage. To ensure the function of arthropod cuticle in protecting the animals from infection and injury, cuticle maintenance and changes in thickness by (re)deposition are likely ongoing processes throughout all stages of the crab life and definitely necessary as part of innate immunity after sudden cuticle injuries as is known from insects [3]. These multiple and fundamental functions in controlling thickening, tanning and maintenance of the cuticle may explain why the presence of Burs with highly conserved sequence is widespread in arthropods and likely indispensible for their successful development and growth.

## Materials and Methods

### Animals

Megalopae and young juvenile blue crabs, *C. sapidus,* were obtained from the blue crab hatchery at the Institute of Marine and Environmental Technology (Baltimore, MD) and were reared in conditions as described [51]. Females at prepubertal stage obtained from a local fisherman were transferred in aerated seawater (10–15 ppt) and kept in similar conditions until pubertal-terminal molt.

### Measurements of Molt-associated Somatic Increment (MSI) and Hardness of Exoskeleton

Cuticle hardness was measured using a hand-held Shore-type durometer (OO gauge, Instron, Grove City, PA, USA; scale 100 = force 1.11 N) from animals (70–100 mm carapace width, CW) at E_100%_ (the completion of ecdysis) to 72 hr after molt, as described [52]. The durometer probe was placed on the mesogastric area of the dorsal carapace for 15 sec. The molt-associated somatic increment (MSI in %) was calculated from the carapace width of animals at various molt stages divided by that of their exuvia.

### Expression of the Recombinant Bursicon Subunits

Two sets of oligonucleotide primers were designed based on the nucleotide sequences of the cDNA encoding Burs α and β. The 5′-end primers contained an *Nco* I site and the 3′-end primers contained an *Eco*R I site. Two PCRs were conducted with the sets of primers using the plasmids containing the Burs α or β cDNA as a template. The amplified cDNA fragments were subcloned into a pGEM®-T Easy vector (Promega). Subsequently, each insert was released from the vector by *Nco* I/*Eco*R I digestion, and then ligated into the *Nco* I/*Eco*R I site of a pET-Duet expression vector (Novagen, USA) separately (Table S1).

*Escherichia coli* Rosetta-gami(DE3)pLysS competent cells (Novagen) were transformed with the expression vector containing the Burs insert, and the transformed cells were selected on LB plates containing ampicillin (70 µg/ml). Bacterial cells from a single colony were grown at 37°C overnight in an LB medium containing kanamycin (70 µg/ml), and then the culture was diluted 50-fold with the same medium. The cultivation was continued at 37°C for 1.5 hr, and then isopropyl-β-D-thiogalactoside (IPTG) was added to the culture at a final concentration of 1 mM. After further culturing for 3 hr, bacterial cells were harvested by centrifugation and suspended in 10% of culture volume of PBS. The cells were disrupted by sonication and centrifuged at 3,000 *g* for 10 min. The supernatant was removed, and the pellet was suspended in PBS. Both the supernatant and the suspended insoluble material were subjected to SDS-PAGE using a 15% gel under reducing conditions that revealed the recovery of both subunits in the insoluble fraction (Fig. S3).

### Chemical Refolding and Purification of the Recombinant Bursicon Heterodimer

Initially, the refolding reactions of the recombinant Burs (rBurs) α and β were performed separately in a redox buffer containing reduced and oxidized forms of glutathione. Each recombinant protein gave one major peak and several minor peaks on RP-HPLC after the refolding reaction. Burs α was rather unstable and a large amount of Burs α formed readily a homodimer. Thus, rBurs α and β were mixed at a ratio of 1∶1 for refolding and dimerization.

Insoluble materials after cell breakage (80 ml-culture equivalent of Burs α and 40 ml culture equivalent of Burs β) were mixed and solubilized in 1 ml of 8 M urea and the refolding procedure was carried as described [53]. In brief, to this solution, 4.9 mg of glutathione (reduced form) and 1 ml of dilution buffer (0.57 M Tris, pH 8.5, containing 11.4% glycerol) were added and stirred gently for 10 min at 4°C. Subsequently, 1 ml each of dilution buffer was added in every 10 min until final volume was reached to 8 ml, and glutathione (oxidized form) was then added to a final concentration of 1 mM. The solution was stirred gently for 3 days at 4°C, and the refolded protein was purified by RP-HPLC on a Gemini column (4.6×150 mm, Phenomenex). Heterodimerized CasBurs was separated as a single peak by RP-HPLC at the following conditions: a 2-min initial holding at 15% acetonitrile in 0.1% TFA, and a 35-min linear gradient of 15–60% acetonitrile in 0.1% TFA at a flow rate of 0.5 ml/min (Fig. S4). In mass spectral analysis of rCasBurs, a molecular ion peak was observed at m/z 26236.0, which was similar to its calculated mass number (26223.5 for (M+H)^+^). Yield was 480 µg per 80 ml culture of α and per 40 ml culture of β. Recombinant CasBurs (1 mg) was used for antibody production (ProteinTech, Chicago).

### Radioimmunoassays (RIAs) for CasBurs and Crustacean Cardioactive Peptide (CCAP)

#### A) Iodination

The rCasBurs and YCCAP iodination was carried as described [27], [54]. ^125^IrCasBurs and ^125^IYCCAP were kept in 50% glycerol at 4°C and used within one or two months after iodination. The specific activity of ^125^IrCasBurs and ^125^IYCCAP ranged from 500 to 600 Ci/mmol.

#### B) Sample preparation: tissue and hemolymph

Animals at various molt stages [55] were placed on ice for 5–10 min prior to tissue and hemolymph collections. Hemolymph was directly withdrawn into a marine anticoagulant [48] at a ratio of 1∶1 (v/v) via the arthrodial membrane between a chela and the first walking leg. Dissected TGC, eyestalk (ES), PO, and brains were immediately frozen on dry ice and stored at −80°C until further use. CasBurs contents of brain, eyestalk ganglia, and PO tissues were estimated after homogenization (Branson Sonifier) in 100 µl of ice-cold PBS and centrifugation at 14,000 rpm at 4°C for 10 min. The supernatants were assayed at the following tissue equivalents: 20–40% of ES, 0.1–0.2% of PO, and 10–20% of brain. TGCs were homogenized in 500 µl of ice-cold DEPC treated PBS, and the samples were equally divided for QRT-PCR analysis and RIA. After repeating the centrifugation step as above, 5–10% of a TGC equivalent was used for CasBurs RIA.

Recombinant CasBurs (2.6 µg) produced in bacteria was eluted from C18 Sep-Pak cartridges (Waters) with 40% and 60% of isopropanol and was exclusively found in the former fraction. After centrifugation at 800 g for 10 min at 4°C, supernatants of hemolymph samples were subsequently extracted in the final concentration of 40% isopropanol (v/v) and centrifuged at 2400 rpm for 20 min at 4°C. The supernatants were recovered in Minisorb tubes (Nunc) and dried by vacuum centrifugation (Jouan). The remaining procedures for sample preparation and detailed RIA were as described [54]. The RIA standards of rCasBurs and CCAP ranged from 0.33 to 330 pM (Figs. S5A, S5B). Production and characterization of the rabbit anti-CCAP serum has been described earlier [25]. A rabbit anti-rCasBurs was produced commercially against the entire recombinant heterodimeric *C. sapidus* bursicon (CasBurs) molecule (Proteintech Group, Chicago). This antiserum did not cross-react with CCAP and vice versa (Figs. S5A, S5B). Final concentrations in the RIA were 1∶2000 for antiCCAP and 1∶1000 for anti-rCasBurs. EC_50_ values of RIAs were 29.1±2.3 pM (n = 8) for rCasBurs and 21.8±1.1 pM (n = 4) for CCAP. Both rCasBurs and CCAP RIAs had the detection limit at ≤2.3 pM.

### RP-HPLC Combined with CasBurs RIA and Western Blotting Analysis

Extracts of POs obtained from juvenile and adult female crabs were separated using RP-HPLC as described above. The absorbance at 210 nm monitored by a photodiode array detector (Fig. 1C). Fractions dried by vacuum centrifugation (Jouan) were analyzed using the CasBurs RIA. Fractions (F) 17 and 21 from chromatographed extracts of juveniles were further separated on an 18% SDS-PAGE and subjected to Western blot analysis as described [56].

### Localization of CasBurs in TGC and POs of Megalopae, Juvenile and Adult Crabs

Whole mount immunohistochemistry was carried out following the procedure as described [57] except using anti-rCasBurs at a final dilution of 1∶1000 in 50 mM phosphate buffer containing 0.5% Triton X (PTX). POs were obtained from 15^th^ crabs at E_1%_ and incubated for 2 days at RT. For pre-absorption controls, 1 µl of anti-CasBurs was incubated with 1 nmol rCasBurs in 10 µl of PBS for 2–7 days at 4°C, prior to use. After washing for 1 day, the sample preparations were incubated with FITC conjugated goat anti-rabbit IgG (Vector labs) at 1∶50–1∶100 diluted in PTX for 2 days at RT. After washing, the preparations were mounted in PTX/glycerol (1∶1, v/v) and examined using a confocal microscope (BioRad). Pictures were obtained from Z-stacked sections. For the double-staining with anti-CCAP and anti-CasBurs, the fixed tissues were incubated in anti-CCAP (final dilution at 2000 fold) and FITC-conjugated goat anti-rabbit IgG at the same dilutions as above. After washing, the samples were incubated with anti-CarCasBurs that was conjugated to Alexa 555 using Zenon® Labeling Technology (Invitrogen, following the manufacturer’s descriptions) at the same dilution.

### rCasBurs Bioassay

Fresh new cuticle with hypodermis was obtained from the mesobranchial area of animals at the following molt stages: D_2_ and E_1%_ were cut into small pieces (5×5 mm) and incubated for 30 min at RT, in the presence of 1 µM rCasBurs that was dissolved in crustacean saline [58]. The control groups were exposed to saline alone or 1 µM CasCHH. Each treatment was triplicated and the incubation was repeated three times. At the end of the incubation, the tissues were briefly rinsed in ice-cold crustacean saline and immediately fixed in 2.5% glutaraldehyde in crustacean saline and stored at 4°C until further processing. Tissues were embedded in Spurr’s epoxy resin [59]; 5–7 sections of 1 µm thickness from each tissue sample were cut on an ultra-microtome (LKB Historange) and stained with 1% toluidine blue in borax buffer. The preparations were examined under a Zeiss standard light microscope, and images were taken with a digital camera and Q Capture program (QImaging).

### Statistical Analysis

All data including QRT-PCR analyses, RIAs, molt increment, shell hardness and cuticle thickness are presented as means ± SEM of the experimental replicates. The analyses of statistical significance were tested using ANOVA followed by the Tukey-Kramer multiple comparison test (GraphPad Instat 3 program, Graphpad).

## Supporting Information

Figure S1 **Multiple sequence alignments of the preprohormones of** ***CasBurs*** **α and β.**
(TIF)

Figure S2 **Co-localization of CCAP and CasBurs in the juvenile crab TGC.**
(TIF)

Figure S3 **SDS-PAGE analysis of rCasBurs α and β.**
(TIF)

Figure S4 **RP-HPLC (Waters Alliance 2695) separation of rCasBurs from a refolding reaction mixture.**
(TIF)

Figure S5 **Standard curves of the rCasBurs and CCAP RIAs.**
(TIF)

Table S1 **Primers for cloning, QRT-PCR assay and the expression of rCasBurs in** ***E. coli.***
(PDF)
