# Supplementary figures and images for: New Functions of Arthropod Bursicon: Inducing Deposition and Thickening of New Cuticle and Hemocyte Granulation in the Blue Crab, Callinectes sapidus
Source: PLoS One. 2012 Sep 28;7(9):e46299. doi: 10.1371/journal.pone.0046299 (PMC3460823; doi:10.1371/journal.pone.0046299)

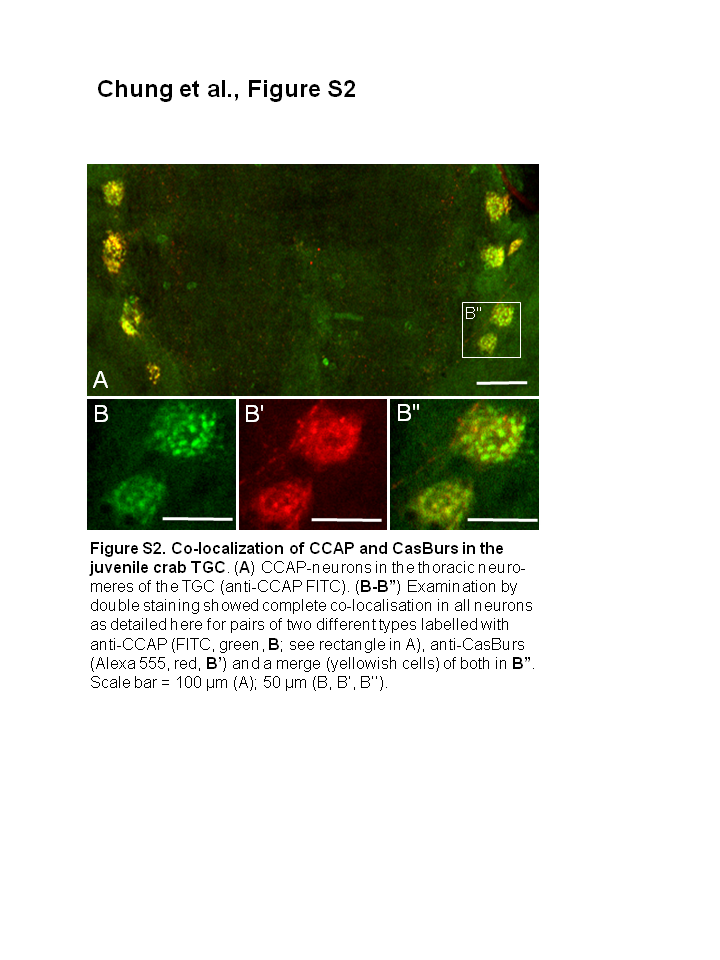

Supplement: Figure S2 — Co-localization of CCAP and CasBurs in the juvenile crab TGC. (TIF) [file pone.0046299.s002.tif]

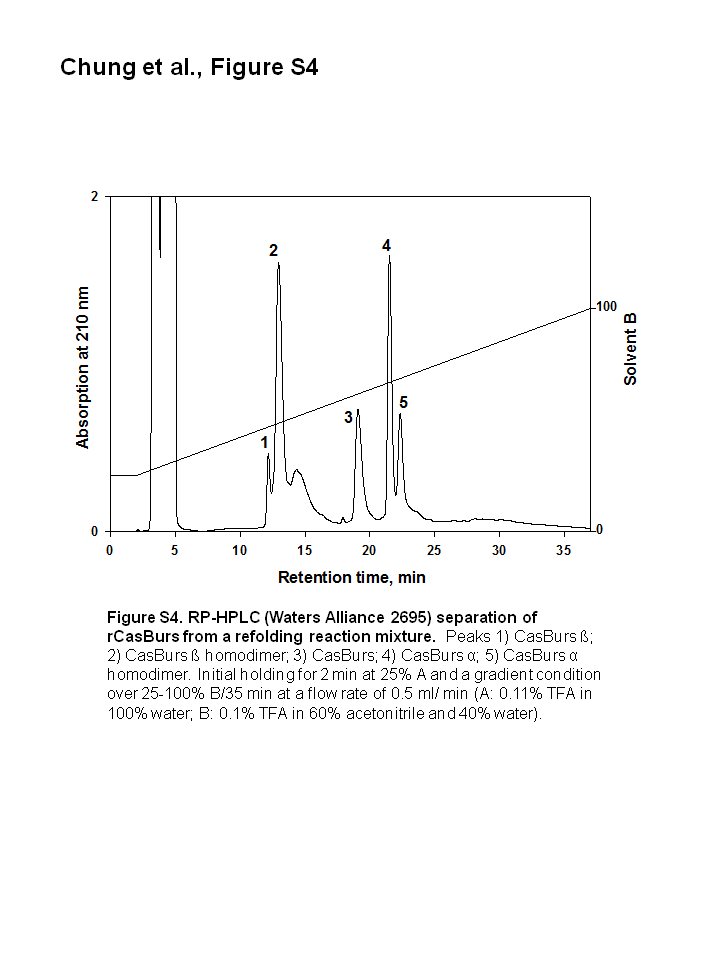

Supplement: Figure S4 — RP-HPLC (Waters Alliance 2695) separation of rCasBurs from a refolding reaction mixture. (TIF) [file pone.0046299.s004.tif]

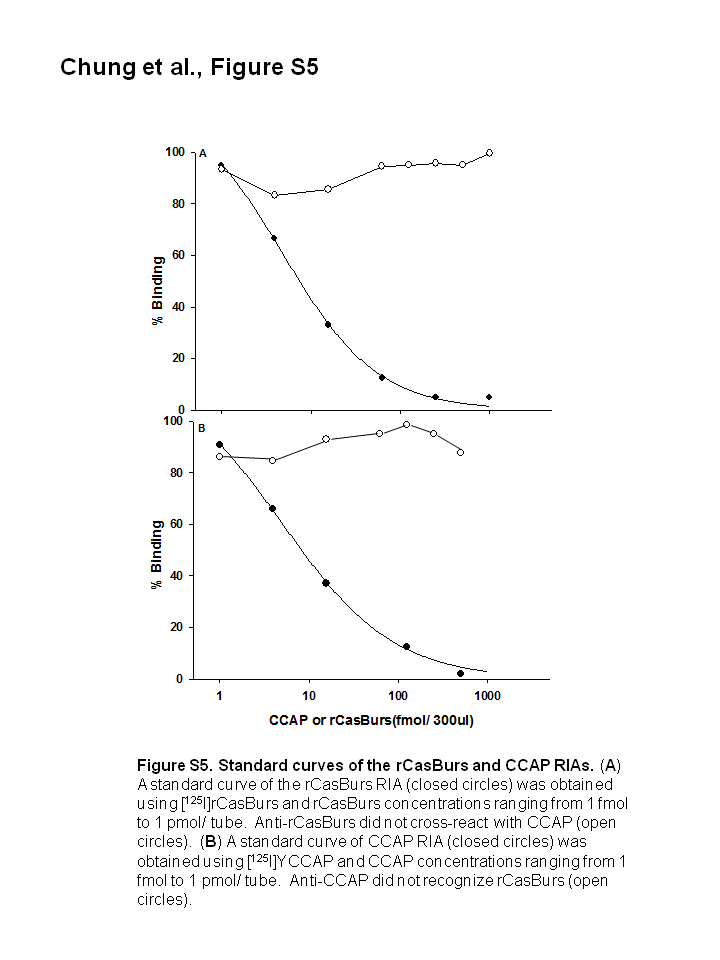

Supplement: Figure S5 — Standard curves of the rCasBurs and CCAP RIAs. (TIF) [file pone.0046299.s005.tif]
