# Supplementary material for: New Functions of Arthropod Bursicon: Inducing Deposition and Thickening of New Cuticle and Hemocyte Granulation in the Blue Crab, Callinectes sapidus
Source: PLoS One. 2012 Sep 28;7(9):e46299. doi: 10.1371/journal.pone.0046299 (PMC3460823; doi:10.1371/journal.pone.0046299)
Supplement: Table S1 — Primers for cloning, QRT-PCR assay and the expression of rCasBurs in E. coli. (PDF) [file pone.0046299.s006.pdf]

Table S1. Primers for cloning, QRT-PCR assay and the expression of rCasBurs in *E. coli*

	Primer sequences (5’-3’)
CasBurs α dF1	CARTAYCCNGGNTGYRTNCC
CasBurs α dF2	TGYTGYCARGARWSNGGNGA
CasBurs α dR1	CACATRCANTCNAVNGGNGC
CasBurs α 5R1	GGCTCTCCGGGGCGCGGCTTG
CasBurs α 5R2	GCGCGGCTTGGGACAGTTGAGGGT
CasBurs α 5R3 (=QR)	TGAAGTTAGCAATCTCCTGGGCA
CasBurs α 3F1	GAGGCGGCTATCACCCTCAACTGTCC
CasBurs α 3F2 (=QF)	CTATCACCCTCAACTGTCCCAAGCCG
CasBurs α NcoI	ATCGCCATGGCCGACGAGTGTTCCCTGCGGCCTGT
CasBurs α EcoRI	ATGAATTCCTACTTCAGAAAGGGAACGCTGTC
CasBurs ß dF1	AARGANGARTAYGAYGARATNGG
CasBurs ß dF2	TGYAAYWSNCARGTNCANCC
CasBurs ß dR1	CCRCAYTTRWARCAYTYRCA
CasBurs ß 5R1	TTCCCTGAGCTTCACCTGCTGGGT
CasBurs ß 5R2 (=QR)	CTGAGCTTCACCTGCTGGGTTGCC
CasBurs ß 3F1	CTCCGTCAACACTCCTTCAGGATT
CasBurs ß 3F2 (=QF)	TCAACACTCCTTCAGGATTCCTCAA
CasBurs ß NcoI	ATCGCCATGGCCAGAACGTACGGTGTTGAATGCGA
CasBurs ß EcoRI	ATGAATTCTTACCGGGTCGAGTCGCCACA
